# Hemodynamic Response to Air-Conducted Sound Stimulus Is Mediated via Vestibulosympathetic Reflex

**DOI:** 10.3390/jcm14196903

**Published:** 2025-09-29

**Authors:** Magdalena Krbot Skorić, Luka Crnošija, Ivan Adamec, Mario Habek

**Affiliations:** 1 School of Medicine, University of Zagreb, 10000 Zagreb, Croatia; mkrbot@gmail.com (M.K.S.); luka.crnosija@gmail.com (L.C.); ivan.adamec@yahoo.com (I.A.); 2 Faculty of Electrical Engineering and Computing, University of Zagreb, 10000 Zagreb, Croatia; 3 Referral Center for Demyelinating Diseases of the Central Nervous System, Department of Neurology, University Hospital Center Zagreb, 10000 Zagreb, Croatia

**Keywords:** air-conducted sound stimulus, autonomic nervous system, respiratory sinus arrhythmia, vestibular neuritis, vestibulosympathetic reflex

## Abstract

**Aim:** To investigate the vestibulosympathetic reflex (VSR) in humans by comparing the hemodynamic responses to air-conducted sound stimulus (ACSS) of the vestibular system between healthy individuals and participants with vestibular neuritis (VN). **Methods:** Twenty-one healthy controls and seven participants with VN were enrolled. Each autonomic test was first conducted without and then with ACSS of the vestibular system. The following autonomic tests were performed: heart rate response to Valsalva maneuver; heart rate response to deep breathing; and heart rate and blood pressure response to a supine position, passive tilt, and active standing. **Results:** In healthy participants, there was a difference between respiratory sinus arrhythmia values without and with otolithic stimulation (26.63 ± 6.16 vs. 24.67 ± 7.34, *p* = 0.02). During passive tilt, the average heart rate throughout ACSS was lower than immediately before ACSS (88.63 ± 14.68 vs. 90.96 ± 14.93, *p* = 0.001). In participants with VN, no such differences were observed. **Conclusions:** This study demonstrated a significant effect of otolithic stimulation with ACSS on heart rate during passive tilt in healthy participants. These findings suggest that ACSS of the vestibular system could be a valuable method for future research on the VSR.

## 1. Introduction

Cardiovascular reflexes help keep blood pressure stable during postural changes. Although the baroreflex is the most well-known reflex governing blood pressure during standing, several studies have also identified a vestibulosympathetic reflex (VSR), which was previously called the vestibulo-autonomic reflex in earlier research [1,2,3,4,5,6,7,8]. This reflex, along with the baroreflexes, works to increase sympathetic nerve activity and control arterial blood pressure during orthostasis [9]. When a person shifts from lying down to standing, 300–800 mL of blood is moved toward the lower extremities by gravity. It has been suggested that the VSR acts even before compensatory mechanisms such as the baroreceptor reflex to counteract the blood pressure drop caused by postural hypotension [10,11].

So far, studies have documented vestibulosympathetic responses in both animals and humans. However, the methods used to test the VSR varied widely across studies, including head-down rotation (HDR), caloric stimulation, yaw head rotation, sinusoidal linear acceleration, off-vertical axis rotation, and galvanic vestibular stimulation (GVS) (Table 1) [3,4,6,7,12,13,14,15,16,17]. Because of these varied approaches, results have been inconsistent, but the most consistent finding is an increase in muscle sympathetic activity (MSNA) in response to HDR (Table 2) [11,12,13,17,18,19,20,21,22,23,24,25,26,27,28,29,30,31,32,33,34]. This rise in MSNA is linked with peripheral vasoconstriction [24]. Several studies have concluded that the VSR produces a stronger effect during states of imminent hypotension [9], indicating that the VSR responds to this threat with peripheral vasoconstriction. Furthermore, numerous studies have observed increases in heart rate and blood pressure following vestibular stimulation (Table 2).

Vestibular evoked myogenic potentials (VEMPs) are another method used to test the vestibular system. The delivery of air-conducted sound stimuli (ACSS), such as clicks or pure tones in the range of 130–145 dB [16,35], activates the saccule and utricle, leading to changes in the electrical potentials (EPs) of the sternocleidomastoid (SCM) and inferior oblique (IOM) muscles, respectively. Changes in EP recorded over the SCM with surface electrodes directly reflect the activity of the afferent branch of the vestibular nerve associated with the saccule (the inferior branch of the vestibular nerve); this is known as cervical VEMP (cVEMP) [8]. Ocular VEMPs (oVEMPs), recorded over the IOM, originate from the utricle and are transmitted via the superior branch of the vestibular nerve [14]. Therefore, VEMPs are valuable tools for assessing the function of the vestibular system across various pathologies. Although ACSS primarily activates the saccule and utricle, which are likely involved in the VSR, this method has never been used to investigate the VSR. Interestingly, studies have explored the VSR using animal models with vestibular lesions [36,37], but no research has actively examined the VSR in human participants with vestibular lesions. Thus, this study aimed to investigate the presence of the VSR in humans by comparing the hemodynamic responses of the vestibular system to ACSS between healthy subjects and participants with vestibular neuritis (VN).

## 2. Methods

### 2.1. Participants

Twenty-one healthy controls and seven participants with VN were included in the study. Of these, five participants with VN had isolated involvement of the superior branch, while two experienced involvement of both the superior and inferior branches of the vestibular nerve. 

Inclusion criteria for participants with VN were: (1) age ≥ 18 years; (2) clinical diagnosis of acute vestibular neuritis, defined by constant rotatory vertigo lasting ≤ 48 h, unidirectional horizontal–rotatory nystagmus beating toward the unaffected ear, positive head impulse test toward the affected ear, and absence of skew deviation (assessed by Maddox rod); and (3) diagnostic confirmation through vestibular testing including caloric test showing vestibular paresis with ≥25% asymmetry (Jongkees’ formula) and/or abnormal video head impulse test (vHIT) and/or abnormal vestibular evoked myogenic potentials (VEMPs). Inclusion criteria for healthy controls included: (1) age ≥ 18 years, (2) no history of vestibular or balance disorders, and (3) normal findings on bedside vestibular examination (head impulse test, absence of spontaneous nystagmus, negative Maddox rod test).

Exclusion criteria (applicable to both participants with VN and controls) included: (1) a history of central nervous system disorders, (2) a history of ear surgery, chronic otitis media, or inner ear malformations, (3) the presence of Meniere’s disease, vestibular migraine, BPPV, or other vestibular disorders, (4) use of vestibular suppressant medication within 48 h before testing, (5) severe cardiovascular, metabolic, or psychiatric illness that interferes with participation, and (6) inability to provide informed consent.

Additionally, all participants underwent a standard clinical otoneurological evaluation, which included bedside hearing assessments such as the finger rub test, whispered voice test, and tuning fork tests (Rinne and Weber). These confirmed that none of the participants had clinically significant hearing impairment [38].

### 2.2. Vestibulosympathetic Reflex Testing

All tests were conducted in a quiet, dimly lit room. Participants were instructed not to drink coffee or smoke before testing. Blood pressure (BP) and heart rate (HR) were recorded using the Task Force Monitor (TFM) (CNSystems Medizintechnik AG, Graz, Austria). After the patient was positioned supine on the testing table, headphones were used to deliver sound stimuli (clicks, referred to as ACSS), while BP cuffs (constant measurement via phalangeal cuff and periodic measurement via brachial cuff) and ECG electrodes were placed at appropriate sites. Once participants were prepared, a 10 min rest period was applied, during which they were in the supine position, to establish baseline cardiac function levels (resting HR and BP). Afterward, ACSS (described in VEMP testing) was administered to the healthy subjects’ right ear at 130 dB for 50 s at a frequency of 1 Hz. Participants with VN received clicks on the affected side at the same intensity and frequency.

The autonomic nervous system testing was conducted as previously described [39]. 

HR response to Valsalva maneuver (3 intervals without ACSS, 2 intervals with ACSS). The Valsalva maneuver was performed in the supine position by blowing through a mouthpiece connected to a mercury manometer for 15 s. The mercury column of the manometer was maintained at 40 mm Hg. There was a small air leak in the system to prevent the glottis from closing. The test was repeated until a reproducible response was observed. The Valsalva ratio (VR) was calculated as the maximum HR during the Valsalva maneuver divided by the lowest HR recorded within 15 s of the peak HR. 

HR response to deep breathing (90 s without, and 50 s with ACSS). Respiratory sinus arrhythmia (RSA) was calculated from the deep breathing exercise. It is determined as the difference between the end of expiration and the end of inspiration in heart rate values (in bpm). An average of at least 5 RSA values is calculated and shown in the results. 

BP response to 70° passive tilt. Participants were tilted for 5 min followed by 50 s of ACSS. The point at which the patient was returned to a supine position is noted.

Lastly, the BP response to 5 min of active standing followed by 50 s of ACSS.

Each test was initially conducted without and then with ACSS of the vestibular system. After each testing session, subjects were given at least 3 min for their heart rates to return to baseline, which was determined during the first 10 min.

### 2.3. Otolithic Stimulation via High-Intensity Air-Conducted Sound

The stimuli (ACSS) were delivered through headphones in a series of 50 trials to the right ear of the healthy subject and the affected ear of the VN patient. VEMP stimuli were delivered using a pair of shielded and calibrated headphones, which are part of the medical device for evoked potentials used in our laboratory [40]. The stimuli consisted of acoustic clicks, 1 ms in duration, at an intensity level of 130 dB SPL. The stimulation rate was 1 Hz. 

### 2.4. Outcomes

The outcomes of the study were to determine differences between parasympathetic measures (VR and RSA) before and during stimulation of the vestibular nerve with acoustic clicks in healthy participants. Additionally, we aimed to examine differences in HR, sBP, and dBP across three positions (supine, tilted, and active standing) with and without vestibular nerve stimulation in healthy participants. Finally, we sought to assess whether these differences, if any, are reduced in participants with vestibular neuritis. 

### 2.5. Statistics

Statistical analysis was conducted using IBM SPSS software, version 20. The Kolmogorov–Smirnov test was used to assess whether the data followed a normal distribution. Differences between variables were evaluated with a paired *t*-test. Bonferroni-adjusted *p*-values were applied in the analysis. 

## 3. Results

### 3.1. Healthy Participants

Twenty-one healthy volunteers participated in the study, including 7 females, with a mean age of 23.53 ± 3.6 years.

There was a statistically significant difference between RSA values without and with otolithic stimulation (26.63 ± 6.16 vs. 24.67 ± 7.34, *p* = 0.02), indicating that RSA values during otolithic stimulation were significantly lower compared to the period without stimulation. There was no statistically significant difference between VR values without and with otolithic stimulation (2.11 ± 0.31 vs. 2.00 ± 0.39, *p* = 0.19). 

Further analysis was performed to compare the average of equal numbers of beat-to-beat values for HR, dBP, and sBP before the ACSS of the vestibule and during stimulation, for the supine position, during the tilt table test, and during active standing.

**Figure 1:**
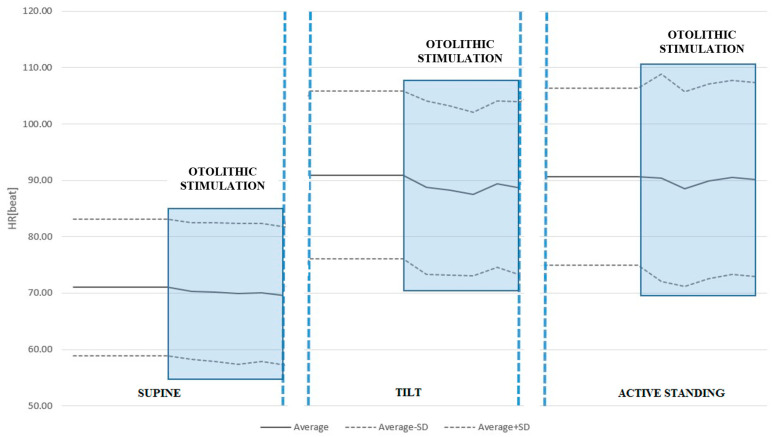
HR response to ACSS of the vestibule during the supine, passive tilt, and active standing positions. Note the significant drop in HR during otolithic stimulation in the tilted position. It should also be noted that passive tilt produces the greatest orthostatic stress.

We found that during passive tilt, the average HR value throughout ACSS of the vestibule was significantly lower than the average HR values immediately before the ACSS (88.63 ± 14.68 vs. 90.96 ± 14.93, *p* = 0.001). There was no statistically significant difference for the supine position and active standing. The results are shown in Table 3 and are graphically presented in Figure 1.

**Figure 2:**
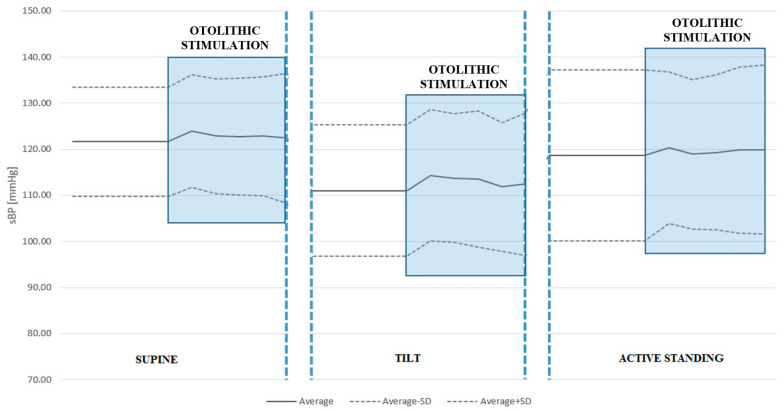
Systolic blood pressure (sBP) response to ACSS of the vestibule during the supine, passive tilt, and active standing positions. Note that the largest increase in sBP was caused by otolithic stimulation during the passive tilt position, although this did not reach statistical significance.

**Figure 3:**
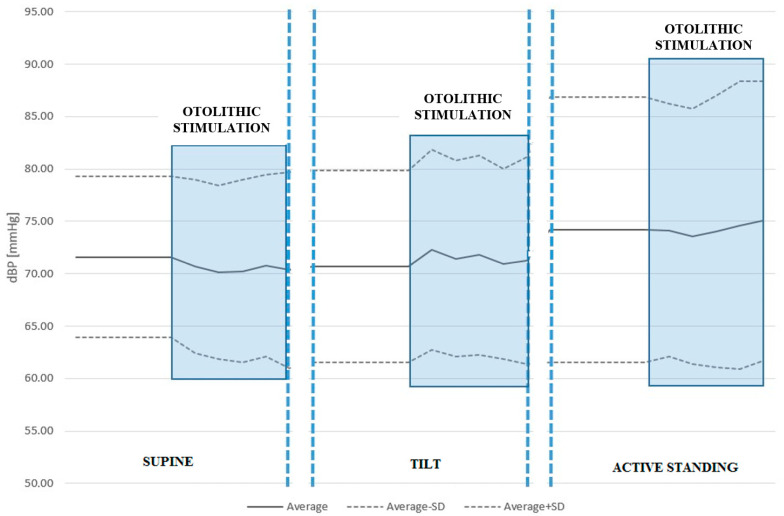
Diastolic blood pressure (dBP) response to ACSS of the vestibule during the supine, passive tilt, and active standing positions. Once again, otolithic stimulation during the passive tilt position caused the largest increase in dBP.

We found no statistically significant differences in sBP or dBP between conditions with and without ACSS of the vestibule in the supine position, during the tilt table test, or active standing. See Table 4 and Table 5 and Figure 2 and Figure 3.

### 3.2. Vestibular Neuritis Participants

Seven participants with vestibular neuritis were included in the study, including four females, with a mean age of 31.14 ± 15.3 years.

In the VN group, we found no statistically significant difference between RSA values without and with otolithic stimulation (25.55 ± 11.8 vs. 24.26 ± 12.48, *p* = 0.356). There was also no statistically significant difference between VR values without and with otolithic stimulation (2.07 ± 0.51 vs. 2.14 ± 0.56, *p* = 0.221). 

Furthermore, there was no statistically significant difference in HR, sBP, or dBP values between conditions with and without otolithic stimulation for the supine position, tilt table test, and active standing. Results are shown in Table 6, Table 7 and Table 8.

## 4. Discussion

The results of this study further strengthen the evidence that the VSR exists in humans. We demonstrated that air-conducted sound stimulation (ACSS) of the otolith organs induces a hemodynamic response, consistent with the theory of the vestibulosympathetic reflex. Although VSR is thought to cause an increase in sympathetic activity, this response was observed as a decrease in heart rate in healthy subjects during passive head-up tilt. These findings supplement previously published results, even though they initially appear to contradict the intended purpose of the VSR, which will be explained further on. The VSR was studied using various protocols (see Table 1 and Table 2), leading to some variability in the results. The most consistent finding was obtained with head-down rotation, which resulted in increased MSNA (Table 2). The activity of sympathetic nerves innervating muscles causes vasoconstriction [41], and it has been repeatedly shown that both MSNA and peripheral vascular resistance increase progressively with the severity of orthostatic stress [42]. One study demonstrated a significantly greater MSNA response to head-down rotation in head-up tilted humans compared to the prone position, indicating that the sensitivity of the VSR is higher in the upright posture [17]. Another study on rats supports this conclusion [36]. Interestingly, in our study, otolithic stimulation caused a statistically significant decrease in HR only during passive head-up tilt, with no significant differences in other hemodynamic parameters during active standing. This may be because passive tilt imposes a higher level of orthostatic stress, as the effect of the skeletal muscle pump on blood pressure regulation is bypassed during passive tilting. Consistent with this, it is argued that the VSR is primarily activated under conditions with a greater risk of hypotension [9]. Still, it seems unusual that activation of the VSR would result in a decrease in HR, since its supposed role is to support the baroreflex in counteracting gravitational blood redistribution during postural changes. Because our subjects first stood in passive head-up tilt for 10 min and then received otolithic stimulation, their cardiovascular systems had sufficient time to adjust. This adjustment is mainly achieved through baroreflex unloading, resulting in decreased parasympathetic activity (cardio-acceleration) and increased sympathetic tone (peripheral vasoconstriction). Although we did not measure MSNA directly, and thus cannot definitively state whether sympathetic activity increased, both the HDR method and ACSS activate the otoliths. It has been repeatedly shown that HDR increases MSNA and causes peripheral vasoconstriction [24]. Since ACSS elicited a hemodynamic response during head-up tilt, we hypothesize that it also caused peripheral vasoconstriction. Notably, both systolic and diastolic blood pressure slightly increased following otolithic stimulation (see Table 4 and Table 5), although these changes did not reach statistical significance. One study indicated that the baroreflex and the VSR have additive effects on blood pressure regulation, suggesting they operate independently [43]. Supporting this, behavioral and cellular physiological studies suggest that the baroreceptor and vestibular reflex pathways remain separate until they synapse on presympathetic neurons in the rostral ventrolateral medulla [44]. Therefore, it is more likely that the decrease in HR during otolithic stimulation in tilted healthy subjects results from baroreflex loading caused by vasoconstriction and blood pressure increases triggered by otolithic stimulation.

In our study, otolithic stimulation was delivered using clicks at a frequency of 1 Hz and an intensity of 130 dB SPL. We acknowledge that vestibular responses, especially those of the otolithic organs, depend heavily on both the frequency and intensity of the acoustic stimulus. This principle is key in clinical applications such as VEMPs, where adjusting stimulus parameters provides diagnostic information: high-intensity stimulation can reveal abnormally low thresholds in superior canal dehiscence [45], while low-frequency stimulation has been associated with specific changes in participants with Ménière’s disease [46].

Our choice of a 1 Hz, 130 dB SPL stimulus was twofold. First, this parameter set has been consistently used in our previous research on vestibular neuritis and persistent postural–perceptual dizziness, ensuring methodological consistency and comparability [47,48]. Second, a high-intensity, low-frequency paradigm was selected to maximize the likelihood of eliciting strong vestibular responses across participants, especially in those with vestibular neuritis, where residual function might be reduced. While this approach improves response reliability, it does not cover the full range of frequency- and intensity-dependent properties of the otolithic organs [49,50]. Therefore, our findings should be interpreted in light of these methodological choices. Future research should examine multiple frequencies and intensities of otolithic stimulation to better understand their effects on the autonomic nervous system.

We also found that heart rate variability in response to paced deep breathing is significantly lower during otolithic stimulation in healthy subjects. The response to the deep breathing test is a common measure of cardiovagal function, and, as in this study, it is typically performed in the supine position where vagal tone is at its highest [51]. Interestingly, otolithic stimulation did not produce any change in HR or blood pressure during the resting period in the supine position, even though the deep breathing test was also performed in this position. This finding might reflect central integration of baroreflex and vestibular input to the rostral ventrolateral medulla [44]. 

Some could argue that sound not only activates vestibular structures but also stimulates the cochlea, and the influence of sound/music on autonomic nervous system regulation of heart activity has been documented [52,53]. Since we observed no statistically significant changes in the parameters studied before and during otolithic stimulation in the VN participants group, these results suggest that a functioning vestibular nerve is necessary to produce a hemodynamic response. This aligns with findings by Abe et al., who observed that rats with vestibular lesions experienced a greater drop in blood pressure upon voluntary rear-up [36]. 

This study has several limitations. The sample was relatively small and non-homogeneous, with variation in both group size and age distribution. This imbalance may have affected the generalizability of our findings. Additionally, assessments were conducted after stabilizing long-arc baroreceptor reflexes; earlier evaluations could have revealed an additive effect of vestibular reflexes, which are known to exert a quicker influence on autonomic responses [54,55].

Nevertheless, this study provides new data on hemodynamic responses to otolithic stimulation in vestibular neuritis. Prior research by [56] documented a higher occurrence of orthostatic hypotension in participants with absent VEMP responses, indicating vestibular dysfunction.

## 5. Conclusions

Our findings show that otolithic stimulation with high-intensity air-conducted sound produces measurable hemodynamic responses in healthy individuals but not in those with vestibular neuritis. This indicates that intact vestibular input is essential for modulating cardiovascular control through the VSR. Clinically, this finding may support using otolithic stimulation protocols as a supplementary tool to assess vestibulo-autonomic function in patients suspected of having vestibular issues. From a diagnostic standpoint, adding such tests to established methods (e.g., VEMPs, caloric testing, vHIT) could improve the characterization of patients with unexplained orthostatic intolerance or chronic dizziness, where autonomic–vestibular interactions might be compromised. Additionally, this approach could eventually aid in early detection of patients at risk for long-term vestibular deficiency or autonomic imbalance after acute vestibular events.

## Figures and Tables

**Table 1:** Methods of vestibular stimulation used in previous studies and vestibular organs they activate.

	Lateral SC	Anterior SC	Posterior SC	Saccule	Utricle
Yaw head rotation (YHR)	+ [11]				
Caloric stimulation [15]	Predominantly	Slightly			
			
Head-down rotation (HDR)	After completion of the rotation influence of semicircular canals is eliminated [15]			+	+
Sinusoidal linear acceleration (SLA)				Predominant activation when supine; slight activation when seated [3]	Predominant activation when seated; slight activation when supine [3]
OVAR [26]	Influence of SCs is eliminated after 10–12 s			+	+
Galvanic stimulation [15]	+	+	+	+	+
VEMP	New evidence raises the possibility of SC activation [26]			+ [13]	+ [13]

+ marks vestibular organs which are stimulated by the particular method. SC—semicircular canal.

**Table 2:** Sympathetic neural and cardiovascular responses to various methods of vestibular system stimulation.

Study	Stimulation Method	MSNA	SSNA	HR	BP
Ray, 1998 [15]	YHR	⇔	⇔	⇔	⇔
Wilson, 2004 [33]	YHR	NS	⇔	NS	NS
Cui, 1999 [21]	Caloric stimulation	NS	⇑ during the first 40 s; ⇓ with the onset of nystagmus	NS	NS
Cui, 1997 [30]	Caloric stimulation	⇑	NS	NS	NS
Costa, 1995 [23]	Caloric stimulation	⇔	NS	⇔	⇔
Short, 1997 [31]	HDR	⇑	NS	⇑	⇑
Wilson, 2004 [33]	HDR	⇑	NS	⇔	⇔
Wilson, 2004 [34]	HDR	NS	⇔	NS	NS
Dyckman, 2007 [24]	HDR	⇑	NS	⇔	⇔
Sauder, 2008 [17]	HDR	⇑	NS	⇔	⇔
Cui, 1999 [22]	SLA	⇓	NS	⇔	⇔
Yates, 1999 [11]	SLA	NS	NS	⇑	⇑
Jaregui-Renaud, 2006 [29]	SLA	NS	NS	⇑	NS
Cui, 2001 [20]	SLA	⇓	NS	⇑ with acceleration peak value of ±20 G; ⇔ with lower accelerations	⇔
Grewal, 2012 [25]	SLA	NS	⇑	⇔	⇔
Hammam, 2013 [12]	SLA	⇑	NS	NS	NS
Hammam, 2014 [26]	SLA	⇑ *	NS	NS	NS
Bolton, 2004 [19]	Galvanic stimulation	⇔	⇑	NS	NS
Voustianiouk, 2006 [32]	Galvanic stimulation	⇑	NS	⇔	⇔
Bent, 2006 [18]	Galvanic stimulation	⇑	NS	NS	NS
Hammam, 2011 [27]	Galvanic stimulation	⇑	NS	NS	NS
Hammam, 2012 [28]	Galvanic stimulation	NS	⇑	NS	NS
Kaufmann, 2002 [13]	OVAR	⇑ when nose-up, ⇓ when nose-down	NS	⇔	NS

The increase in MSNA during head-down rotation is a consistent finding and has been replicated in more than 10 studies not enlisted here. * In this study, patients underwent SLA in supine and seated position, thus activating both the saccule and utricle, respectively. Other studies listed here employed SLA with the patient seated, thus predominantly activating the utricle. YHR—yaw head rotation, HDR—head-down rotation, SLA—sinusoidal linear acceleration, OVAR—off-vertical axis rotation, HR heart rate, BP—blood pressure (i.e., systolic, diastolic or mean arterial pressure), NS—not studied, ⇑—increase, ⇓—decrease, ⇔—no change.

**Table 3:** Comparison of HR values of healthy subjects between condition with and without otolithic stimulation during supine position, tilt table test and active standing.

Position	Otolithic Stimulation	Mean HR (bpm)	Std. Deviation	Median HR (bpm)	Sig. (2-Tailed)
Supine	−	70.99	12.20	70.82	NS
	+	70.07	12.16	68.55	
Tilt	−	90.96	14.93	94.73	0.001
	+	88.63	14.68	90.55	
Active standing	−	90.79	15.78	88.73	NS
	+	90.02	17.17	90.19	

**Table 4:** Comparison of sBP values of healthy subjects between condition with and without otolithic stimulation during supine position, tilt table test and active standing.

Position	Otolithic Stimulation	Mean sBP (mmHg)	Std. Deviation	Median sBP (mmHg)	Sig. (2-Tailed)
Supine	−	121.76	11.70	121.32	NS
	+	122.93	12.67	124.93	
Tilt	−	111.17	14.15	108.09	NS
	+	113.16	14.16	111.84	
Active standing	−	118.92	18.41	112.68	NS
	+	119.63	17.03	116.33	

**Table 5:** Comparison of dBP values of healthy subjects between condition with and without otolithic stimulation during supine position, tilt table test and active standing.

Position	Otolithic Stimulation	Mean dBP (mmHg)	Std. Deviation	Median dBP (mmHg)	Sig. (2-Tailed)
Supine	−	71.64	7.67	71.08	NS
	+	70.43	8.47	69.60	
Tilt	−	70.70	9.17	69.09	NS
	+	71.55	9.24	69.08	
Active standing	−	74.59	12.94	73.53	NS
	+	74.31	12.75	73.67	

**Table 6:** Comparison of HR values of VN patients between condition with and without otolithic stimulation during supine position, tilt table test and active standing.

Position	Otolithic Stimulation	Mean HR (bpm)	Std. Deviation	Median HR (bpm)	Sig. (2-Tailed)
Supine	−	78.16	9.9	79.18	NS
	+	74.85	7.85	76.06	
Tilt	−	91.57	15.91	84.52	NS
	+	93.19	15.33	88.68	
Active standing	−	99.13	16.62	93.24	NS
	+	97.81	15.87	94.88	

**Table 7:** Comparison of sBP values of VN patients between condition with and without otolithic stimulation during supine position, tilt table test and active standing.

Position	Otolithic Stimulation	Mean sBP (mmHg)	Std. Deviation	Median sBP (mmHg)	Sig. (2-Tailed)
Supine	−	116.18	8.29	115.30	NS
	+	118.66	8.32	119.66	
Tilt	−	106.41	12.30	104.48	NS
	+	109.50	10.17	104.94	
Active standing	−	120.65	15.46	119.62	NS
	+	120.40	14.41	120.80	

**Table 8:** Comparison of dBP of VN patients values between condition with and without otolithic stimulation during supine position, tilt table test and active standing.

Position	Otolithic Stimulation	Mean dBP (mmHg)	Std. Deviation	Median dBP (mmHg)	Sig. (2-Tailed)
Supine	−	73.92	4.94	74.56	NS
	+	73.98	4.38	75.86	
Tilt	−	73.13	11.00	72.43	NS
	+	74.10	8.24	72.12	
Active standing	−	76.64	13.14	76.19	NS
	+	76.57	13.05	77.50	

## Data Availability

The data supporting this study’s findings are available from the corresponding author upon reasonable request.

## Abbreviations

ACSS, air-conducted sound stimulus; BP, blood pressure; EPs, electrical potentials; GVS, galvanic vestibular stimulation; HDR, head-down rotation; HR, heart rate; IOM, inferior oblique muscles’; MSNA, muscle sympathetic activity; RSA, respiratory sinus arrhythmia; SCM, sternocleidomastoid; VSR, vestibulosympathetic reflex; VEMPs, vestibular evoked myogenic potentials; VN, vestibular neuritis; VR, Valsalva ratio.
